# Recovery of BMD after pregnancy and breastfeeding—a 10-yr prospective observational study of 25-yr-old women

**DOI:** 10.1093/jbmr/zjaf087

**Published:** 2025-06-24

**Authors:** Lisa Egund, Linnea Malmgren, Anthony D Woolf, Fiona E McGuigan, Kristina E Akesson

**Affiliations:** Lund University, Orthopedics, Department of Clinical Sciences Malmö, 205 02 Malmö, Sweden; Skåne University Hospital, Department of Orthopedics, 205 02 Malmö, Sweden; Lund University, Orthopedics, Department of Clinical Sciences Malmö, 205 02 Malmö, Sweden; Skåne University Hospital, Department of Orthopedics, 205 02 Malmö, Sweden; Skåne University Hospital, Department of Geriatrics, 205 02 Malmö, Sweden; Lund University, Orthopedics, Department of Clinical Sciences Malmö, 205 02 Malmö, Sweden; Lund University, Orthopedics, Department of Clinical Sciences Malmö, 205 02 Malmö, Sweden; Skåne University Hospital, Department of Orthopedics, 205 02 Malmö, Sweden; Lund University, Orthopedics, Department of Clinical Sciences Malmö, 205 02 Malmö, Sweden; Skåne University Hospital, Department of Orthopedics, 205 02 Malmö, Sweden

**Keywords:** BMD, lactation, parity, women, bone loss

## Abstract

Pregnancy and lactation require large amounts of calcium, potentially depleting the young-adult bone. This study investigated BMD and fluctuations of BMD resulting from parity and lactation in the PEAK-25 cohort, a prospective observational study of women all aged 25 at inclusion and 35 at follow-up. The analyses used women who were nulliparous at baseline and parous (*n* = 573) or nulliparous (*n* = 177) 10 yr later. Parity, regardless of number of pregnancies, had no negative impact; indeed, spine BMD at age 35 was higher (2.1%; *p* = .043). Likewise, BMD did not differ in women who breastfed, were nonlactating or nulliparous. Even the cumulative duration of breastfeeding did not make a difference. Overall, regardless of parity, in the cohort, by age 35 BMD was already decreasing, with overall losses at the FN (∆, -3.4%) and TH (∆, -2.7%), although not the spine (∆, 0.9%). Yet, BMD fluctuations associated with pregnancy, lactation, and weaning were seen in the short term. Comparing those pregnant >24 mo with those <24 mo prior to DXA, BMD was lowest in women more recently pregnant (FN, -2.2%, TH -2.7%). Women pregnant within 12 mo had 4% lower TH BMD compared with more than 36 mo (*p* = .054, *p*_adjusted_ = .032). Cumulative duration of breastfeeding was associated with bone loss, particularly beyond 15 mo (FN: ∆, -4.3%; TH: ∆, -3.7%) and lower spine BMD accretion. Despite such periods of loss, BMD recovers, evidenced by time-from-weaning to DXA. Women weaning within 6 mo of measurement had lower FN BMD than those where the interval was >24 mo (6.6% vs 1.7%; *p* < .001). In conclusion and despite repeated fluctuations in BMD resulting from the physiological demands of multiple pregnancies and periods of breastfeeding, BMD recovers and ultimately does not differ from that of identically aged women without children.

## Graphical Abstract

**Graphical Abstract:**
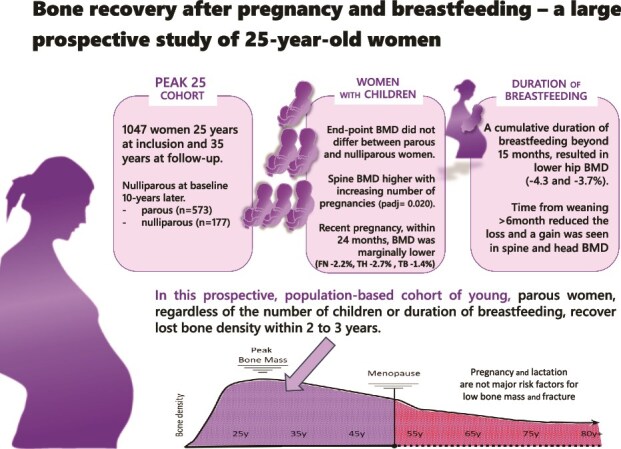

## Introduction

A major determinant of osteoporosis and fracture risk in later life is the maximal amount of bone accrued by the end of skeletal development^1^ and, subsequently, the amount of bone lost with age. Influenced by sex hormone exposure, genetics, and a multitude of lifestyle factors, including diet, smoking, and physical activity, peak bone mass is reached between the ages of 20 and 30. This period is also the time when many women have their first pregnancy.

Pregnancy and lactation require large amounts of calcium and may pose a threat to the maternal “bone bank.” By the end of gestation the human fetal skeleton has accreted approximately 30 g of calcium, with the greatest demand during the third trimester, corresponding to 300-350 mg daily between the 35th and 40th week of gestation. To meet this demand, intestinal absorption of calcium increases. Correspondingly, since fetal calcium demand does not increase until the third trimester, absorptive hypercalciuria is also present. Additionally, in the third trimester, resorption from the maternal skeleton may contribute to a small degree, increasing in case of low calcium intake in the mother. During lactation the main mechanism switches to renal calcium conservation and increased calcium resorption from the maternal skeleton.^2^^,^^3^

Thus, pregnancy and breastfeeding may affect the skeletal integrity of the mother. Naturally, studies on BMD changes during pregnancy are few since fetal radiation expose is preferably avoided, while it may be difficult to distinguish the effect of pregnancy from that of breastfeeding in studies investigating BMD shortly after pregnancy. Notwithstanding, some small studies indicate that pregnancy may have a negative short-term effect on BMD, depending on site.^2^^,^^4^^,^^5^ This may also be the case with lactation. A systematic review from 2021 investigating the effect of lactation on BMD included 21 studies of women aged 23-42 yr and, although all but one was prospective, the sizes were small (5-132 participants) and few used DXA beyond 12 mo postpartum. Nevertheless, this study concluded that BMD decreases during pregnancy and lactation, recovering after breastfeeding ceased.^6^ Correspondingly, another review suggests a temporary loss of BMD at the LS after 4-6 mo of breastfeeding.^7^ Both studies emphasized the need for further prospective investigations. Data on long-term effects on BMD are more reliable, with 2 large prospective studies in postmenopausal women concluding that parity and lactation have no adverse effect on BMD in later life,^8^^,^^9^ although it is possible this conclusion may not be generalized to groups with poor nutritional intake.^10^^,^^11^

Subsequently, and to provide better evidence for young-adult women considering pregnancy and breastfeeding, we identified the need for a large prospective study in women of reproductive age, near peak bone mass, and for a longer time period. Aiming to address this gap in knowledge, this study analyzes women from the PEAK-25 cohort, a prospective observational study of over 1000 women all aged 25 yr at inclusion and 35 yr at follow-up. The overall aim was to investigate the association between completed pregnancies, breastfeeding, and bone density at age 35 compared with those who never had children. Specifically, we aimed to explore how the “endpoint” BMD of women at age 35 is influenced by parity and lactation. With this objective, we evaluated bone loss, the extent to which BMD recovers, as well as the length of time it takes to recover at different skeletal sites.

## Materials and methods

### Participants

**Figure 1:**
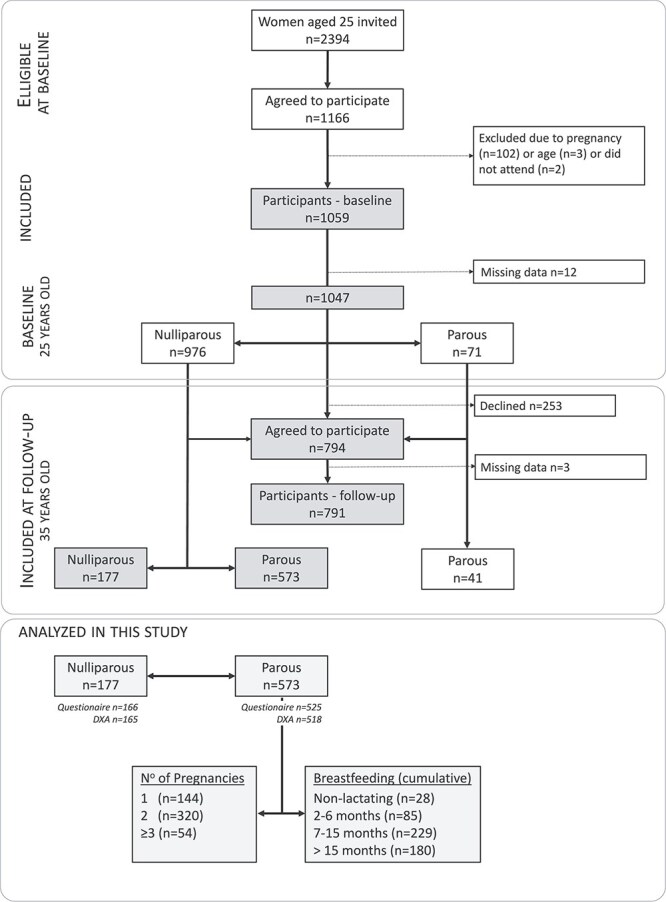
PEAK-25 cohort study: design and data availability for this study of pregnancy and breast feeding and the association with bone density.

The PEAK-25 cohort is a longitudinal, prospective, population-based study of young-adult women with the main purpose of exploring the relationship between genetic, clinical, and lifestyle factors associated with attainment of peak bone mass^12^ and subsequent changes in bone mass over time. Briefly, during 1999-2004, 25-yr-old women from Malmö, Sweden, were invited to participate. Criteria for inclusion were being age 25 yr and female; the criterion for exclusion was pregnancy (current or within 12 mo). A total of 2394 women were invited, and 1166 agreed to participate. The final number at inclusion was 1047 women (exclusions due to 102 who were pregnant, 3 who were outside age limit, 2 did not attend, and 12 were missing data) (Figure 1).

All participants were invited to a follow-up investigation after approximately 10-yr (ie, age 35), of whom 794 (75% response) participated in the investigation (253 declined). Women who were pregnant/breastfeeding attended when possible, hence the time range between visits (mean, 10.9 ± 0.9 yr). Some who did not attend (moved out of area/abroad) completed the questionnaire (*n* = 70). Three cases had missing data, leaving 791 participants in the cohort. Overall, there were no anthropometric and lifestyle differences between women attending follow-up and those who did not, with the exception of BMI, which was higher in non-attenders (23.7 vs 22.8; *p* = .002) (Table S1).

All parts of the study were approved by the Lund University Ethics Review Board (DNR LU 280-99 and LU 567/2008) and the Swedish Data Inspection Board and was performed in compliance with the Helsinki Declaration. Participants were informed of the purpose of the study and gave their signed informed consent before being enrolled.

### Data collection

Information was self-reported, collected by questionnaire at baseline and follow-up. Data included the number of completed pregnancies (parity), dates of each birth, and duration of breastfeeding for each child. Data were collected on menstruation cycle, reproductive difficulties, diet, smoking, alcohol, physical activity, and general health status, including conditions associated with low bone density (inflammatory bowel disease, gluten intolerance, rheumatoid arthritis, cancer, or taking cortisone tablets for ≥3 mo). Anthropometric characteristics were measured at the time of BMD measurement.

### Bone mineral density

Bone mineral density (g/cm^2^) at the FN, TH, LS (L2-L4), total body (TB), and head was measured at inclusion and follow-up using DXA (Lunar Prodigy, software versions 2.15-7.70; GE Healthcare Lunar, Madison, Wisconsin, USA) and performed by the same technicians. Stability and accuracy were monitored using a manufacturer-supplied phantom 3 times/wk; precision coefficients (CV%) have been reported (0.9% FN, 0.5% TH, and 0.7% LS).^12^

### Quantitative ultrasound

Quantitative ultrasound was measured at the calcaneus by the same technicians (Achilles Insight, GE Medical systems, USA). Variables include speed of sound (SOS; m/s), broadband ultrasound attenuation (BUA; dB/MHz), and stiffness index (SI). Daily calibrations were performed: CV 1.5%.^13^

### Study design

The inclusion of more than 850 participants in the PEAK-25 cohort was estimated to be a sufficient sample size to detect differences in BMD. Based on the assumption of an SD of 0.13 g/cm^2^ in BMD, differences of 0.056 g/cm^2^ between decentiles should be detectable (alpha, 0.05; beta, 0.80).

### Type of study

This was a human observational cohort study. The STROBE (Strengthening the Reporting of Observational Studies in Epidemiology) guidelines were applied to ensure transparency in reporting of the study design, analyses, and results.

### Statistical analyses

The primary analyses are based on *n* = 683 women who were nulliparous at baseline and subsequently parous or nulliparous at follow-up (ie, completed pregnancy or not between age 25 and 35) (Figure 1). Those with pregnancies prior to baseline are not included in the main analysis. The endpoint BMD is at age 35 yr. Descriptive data are based on information from all participants who completed the questionnaire at follow-up. Statistical comparisons are based only on women with both DXA and questionnaire data available at follow-up.

Quantitative data were assessed for normality (Kolmogorov-Smirnov test, Q-Q plots, and histograms). There were no outliers suggestive of pregnancy- and lactation-induced osteoporosis. Categorical variables are expressed as number (%) and continuous variables as mean (SD) and/or median (range; IQR). Comparison of continuous variables between 2 groups used an independent unpaired *t* test, and chi-square test for categorical variables. Comparison across multiple variables used ANOVA with post hoc analysis. Linear regression analysis of absolute BMD values was performed, and data adjusted for BMI, smoking, alcohol, and physical activity level. Additional adjustment for the number of pregnancies was performed, where relevant.

To explore the association between parity and bone mass, women were categorized as parous or nulliparous at follow-up (ie, completed pregnancy or not between age 25 and 35). Parous women were further categorized as having 1 pregnancy, 2 pregnancies, or more than 3 completed pregnancies. The time from last pregnancy until DXA was also calculated.

To explore the association between breastfeeding and bone mass, the cumulative duration of breastfeeding was calculated; then, women were categorized as follows: 0-1 mo (non-lactating) and 2-6, 7-15, and more than 15 cumulative months of breastfeeding (breastfeeding <2 mo is unlikely to affect bone mass). Adjustment included the number of completed births. The time from weaning until DXA was also calculated. Change in BMD between ages 25 and 35 was calculated individually for each woman. Data are reported as ΔBMD and %ΔBMD.

To investigate the trajectory of BMD change over 10 yr in young-adult women, we analyzed the complete cohort. To capture alterations in the trajectory attributable to childbearing we analyzed BMD change based on parity, number of pregnancies (1, 2, ≥3), and cumulative duration of breastfeeding (0-1, 2-6, 7-15, >15 mo).

To investigate time effects on BMD recovery, we analyzed the time between weaning and DXA (<6, 7-12, 13-24, and > 24 mo) and time from last pregnancy to DXA (>24 or < 24 mo; and categories ≤12, 13-36, and >36 mo), the outcome being BMD change.

All analyses were performed using SPSS version 29 software (SPSS Inc., Chicago, IL, USA). A 2-tailed *p* value ≤ .05 was considered nominally statistically significant.

## Results

PEAK-25 participants were aged 25 yr at baseline and 35 yr at follow-up (Table 1). At follow-up, 76% (573/750) of the women were parous, having completed at least 1 pregnancy during the intervening 10 years (Figure 1). BMD at baseline and follow-up for the complete cohort is reported in Table 2A. Between the ages of 25 (when all women were nulliparous) and 35 (regardless of parity), BMD at the FN and TH was lower, while BMD was higher at the LS.

**Table 1:** PEAK-25 cohort participant characteristics in the women who were nulliparous at baseline and subsequently nulliparous or parous at follow-up.

	**Nulliparous at age 25 (*n* = 976)**	**Nulliparous at age 35 (*n* = 166)**	**Parous at age 35 (*n* = 525)**
**Age, yr**	25.5 ± 0.2	36.3 ± 0.7	36.5 ± 0.8
**Height, cm**	167.4 ± 6.1	167.8 ± 6.5	167.9 ± 6.0
**Weight, kg**	64.5 ± 11.1	71.5 ± 15.8	68.5 ± 12.2
**BMI, kg/m** ^**2**^	22.9 ± 3.7	25.4 ± 5.4	24.3 ± 4.2
**Educational level—primary/secondary**	577 (61%)	60 (34%)	161 (28%)
**Educational level—university**	384 (39%)	117 (66%)	412 (72%)
**Smoker—current**	244 (25%)	25 (14%)	31 (5%)
**Smoker—non/previous**	729 (75%)	152 (86%)	541 (95%)
**Alcohol, median (IQR), g/wk**	32 (16;56)	29 (14;60)	28 (14; 56)
**Physical activity—high**	512 (53%)	99 (56%)	254 (44%)
**Menstruation cycle—abnormal** ^**a**^	162 (17%)	24 (14%)	71 (13%)
**Reproductive difficulties**	—	29 (16%)	103 (18%)
**Conditions associated with osteoporosis** ^**b**^	47 (5%)	25 (16%)	47 (10%)
**Eating disorder**	—	24 (16%)	49 (11%)
**Dietary calcium intake,** ^**c**^ **mg**	567 ± 338	505 ± 286	533 ± 281
**No. of completed pregnancies**			
**1**	—	—	156 (27.2%)
**2**	—	—	346 (60.4%)
**3 or more (3-5)**	—	—	71 (12.4%)
**Total no. of children (mean *n*)**	—	—	1066 (1.9)
**Time from last pregnancy to DXA (median), mo**	—	—	32 (15-54)
**Breastfeeding, % (*n*/total *n*)**	—	—	94% (538/573)
**Cumulative months of breastfeeding**	—	—	14 (2-56)
**Time from weaning to DXA (median), mo**	—	—	25 (9-48)

Based on all participants completing a questionnaire, age 35 yr. Values are reported as mean (SD), *n* (%), median (range), mean (range).

^a^Irregular or skipping >1 periods/yr.

^b^Inflammatory bowel disease, gluten intolerance, rheumatoid arthritis, cancer, or taking cortisone tablets ≥3 mo.

^c^Daily calcium intake from milk, cheese, or yogurt.

**Table 2:** BMD in the PEAK-25 cohort (A) in all participants and (B) in the group of parous and nulliparous women.

	**A. BMD in the PEAK-25 cohort** ^**a**^ **at ages 25 and 35**				**B. Endpoint BMD (age 35) in parous/nulliparous women** ^**b**^		
	**BMD age 25 in the cohort, nulliparous (NP) age 25** ^**b**^ **(*n* = 683)**	**BMD age 35 in the cohort, all women (parous [P] + NP) age 35** ^**b**^ **(*n* = 518 + 165)**	**%ΔBMD**	***p* value, NP**_**25**_ **vs All**_**35**_	**Parous at age 35 (*n* = 518)**	**Nulliparous at age 35 (*n* = 165)**	***p* value (*p*-adj), P**_**35**_ **vs NP**_**35**_
**DXA BMD (g/cm** ^**2**^ **)**							
**FN**	1.056 (0.12)	1.016 (0.12)	−3.4 (6.1)	<.001	1.017 (0.12)	1.013 (0.14)	.725 (.198)
**TH**	1.063 (0.12)	1.031 (0.12)	−2.7 (0.6)	<.001	1.033 (0.12)	1.025 (0.13)	.481 (.070)
**Spine**	1.239 (0.13)	1.249 (0.14)	0.9 (4.7)	<.001	1.256 (0.13)	1.229 (0.15)	**.040 (.004)**
**Total body**	1.174 (0.07)	1.175 (0.09)	0.0 (3.3)	.614	1.174 (0.09)	1.181 (0.10)	.396 (.924)
**Head**	2.302 (0.27)	2.470 (0.27)	6.7 (4.4)	<.001	2.476 (0.27)	2.461 (0.27)	.572 (.714)
**T-score**			**ΔT-score**				
**FN**	0.632 (1.03)	−0.159 (0.90)	0.79 (0.56)	<.001	−0.15 (0.87)	−0.18 (0.87)	.725 (.198)
**TH**	0.523 (1.03)	0.182 (0.97)	0.34 (0.52)	<.001	0.20 (0.95)	0.12 (1.04)	.357 (**.036**)
**LS**	0.325 (1.11)	0.411 (1.17)	0.09 (0.53)	<.001	0.47 (1.12)	0.24 (1.29)	**.043 (.004)**
**QUS**	*n* = 683	*n* = 683			*n* = 394	*n* = 128	
**SOS (m/s)**	1577 (33)	1585 (35)	—		1586 (33)	1584 (38)	.747 (.441)
**BUA (dB/MHz)**	117.6 (10.5)	115.9 (16.0)	—		115.4 (15.2)	117.7 (18.3)	.196 (.676)
**SI**	99.7 (14.6)	101.3 (17.6)	—		101.0 (16.5)	102.4 (20.7)	.470 (.996)

Values are reported as mean (SD). *p* values across all groups derived by independent *t* test; adjusted for BMI, alcohol, smoking, and physical activity by linear regression. *p* < .05 considered nominally significant and highlighted in bold.

^a^Women who participated in both investigations and had complete data on pregnancies and DXA.

^b^Nulliparous or parous at follow-up. Those with pregnancies prior to baseline are not included.

Abbreviations: BUA, broadband ultrasound attenuation; SI, stiffness index; SOS, speed of sound.

### Pregnancy, parity, time from pregnancy, and endpoint BMD

Endpoint BMD at the FN, TH, and TB did not differ between women who had completed at least 1 pregnancy during the study period and those who were nulliparous (Table 2B). In contrast, parous women had higher spine BMD (2.1%; *p* = .043; *p*_adjusted_ [*p*_adj_] = .004).

The majority had completed 2 or more pregnancies (Table 1), but regardless of number (0, 1, or more), there were no differences in endpoint BMD at the FN, TH, and TB (Table 3). Spine BMD was higher with increasing number of pregnancies (*p*_adj_ = .020) compared with nulliparous women and also reflected in the T-score (*p*_adj_ spine = .003). Multiparous women showed a tendency towards higher BMD, with post hoc analysis indicating the difference lay between the group with 3 or more pregnancies and the nulliparous group (TH, *p* = .019).

**Table 3:** Number of pregnancies and endpoint BMD (age 35) in nulliparous, parous, and multiparous women.

**BMD at age 35 (site)**	**Nulliparous (*n* = 165)**	**1 Pregnancy (*n* = 144)**	**2 Pregnancies (*n* = 320)**	**≥3 Pregnancies (*n* = 54)**	***p* value (*p*-adj)**
**DXA BMD (g/cm** ^**2**^ **)**					
**FN**	1.013 (0.14)	1.012 (0.13)	1.018 (0.12)	1.023 (0.10)	.926 (.321)
**TH**	1.025 (0.13)	1.032 (0.13)	1.031 (0.12)	1.048 (0.10)	.699 (.106)
**LS**	1.229 (0.15)	1.254 (0.14)	1.256 (0.13)	1.265 (0.13)	.172 (**.020**)
**Total body**	1.181 (0.10)	1.168 (0.10)	1.173 (0.90)	1.190 (0.08)	.363 (.092)
**Head**	2.461 (0.27)	2.431 (0.25)	2.482 (0.28)	2.560 (0.25)	**.020 (.011)**
**T-score**					
**FN**	−0.18 (0.97)	−0.19 (0.91)	−0.14 (0.87)	−0.11 (0.75)	.926 (.067)
**TH**	0.12 (1.04)	0.19 (1.06)	0.19 (0.93)	0.32 (0.78)	.624 (**.009**)
**LS**	0.25 (1.29)	0.45 (1.18)	0.47 (1.10)	0.41 (1.17)	.172 (**.003**)
**QUS**	*n* = 128	*n* = 144	*n* = 320	*n* = 54	
**SOS (m/s)**	1584 (38)	1589 (32)	1586 (34)	1577 (30)	.261 (.168)
**BUA (dB/MHz)**	117.7 (18.3)	118.5 (15.8)	114.5 (15.0)	113.4 (13.9)	.066 (.153)
**SI**	102.4 (20.7)	103.6 (17.2)	100.5 (16.3)	97.4 (15.0)	.200 (.238)

Values are reported as mean (SD). *p* values across all groups derived by ANOVA. *p* values adjusted for BMI, alcohol, smoking, physical activity. *p* < .05 was considered nominally significant and highlighted in bold. Those with pregnancies prior to baseline are not included in these analyses.

Abbreviations: BUA, broadband ultrasound attenuation; SI, stiffness index; SOS, speed of sound.

The median time from the last completed pregnancy to the DXA investigation was 32 mo (IQR, 15-54 mo). Comparing those who had been pregnant more than 24 mo with those less than 24 mo prior to DXA, BMD was lower in women who had been more recently pregnant, although absolute differences were marginal (FN, -2.2%; TH, -2.7%; and TB, -1.4%). The result remained after adjustment for TH and TB.

Women who had been pregnant within 12 mo prior to DXA had the lowest values, with 4% lower BMD at the TH compared with those whose pregnancy was at more than 36 mo (*p* = .054, *p*_adj_ = .032). At the spine or TB there were no apparent differences regardless of time from last completed pregnancy to DXA.

Parous women had a lower BMI compared with nulliparous women (Table 1) (*p* = .021) and BMI was lower in women with more children (1 child: BMI, 25.1 kg/m^2^; 2 children: BMI, 24.1 kg/m^2^; and ≥3 children: BMI, 23.3 kg/m^2^; *p* = .009). However, there were no significant differences in change in BMI over time between parous and nulliparous women (BMI units, 1.9 vs 2.0), although the change in BMI (weight gain) was also lower with increasing number of children (1 child: BMI unit, 2.1; 2 children: 1.6; and ≥3 children: 1.0; *p* < .001).

### Breastfeeding and endpoint BMD

The majority of women breastfed, on average, 8 mo per child (range, 2-39 mo). BMD did not differ between women who breastfed or were non-lactating (Table 4). In addition, compared with the nulliparous women, the only observed difference was a slightly higher BMD at the spine (1.256 vs 1.229 g/cm^2^; *p* = .045).

**Table 4:** Duration of breastfeeding and endpoint BMD (age 35).

	**Cumulative duration of breastfeeding (*n* = 562)**				
**BMD at age 35 (site)**	**Non-lactating** ^**a**^ **(*n* = 32)**	**2–6 mo (*n* = 95)**	**7-15 mo (*n* = 240)**	**>15 mo** ^**a**^ **(*n* = 195)**	**Overall *p* value (*p*-adj)**
**DXA BMD (g/cm** ^**2**^ **)**					
**FN**	1.032 (0.11)	1.022 (0.12)	1.015 (0.12)	1.015 (0.12)	0.882 (0.969)
**TH**	1.063 (0.11)	1.040 (0.12)	1.029 (0.13)	1.030 (0.11)	0.517 (0.789)
**LS**	1.245 (0.11)	1.252 (0.15)	1.256 (0.14)	1.261 (0.13)	0.917 (0.502)
**Total body**	1.128 (0.07)	1.175 (0.09)	1.167 (0.09)	1.174 (0.09)	**0.045** (0.263)
**Head**	2.599 (0.23)	2.448 (0.27)	2.456 (0.29)	2.498 (0.25)	**0.028** (0.263)
**T-score**					
**FN**	−0.04 (0.77)	−0.12 (0.90)	−0.17 (0.89)	−0.16 (0.86)	0.882 (0.703)
**TH**	0.43 (0.89)	0.26 (0.94)	0.17 (1.00)	0.19 (0.90)	0.575 (0.360)
**LS**	0.38 (0.92)	0.43 (1.22)	0.47 (1.14)	0.51 (1.08)	0.917 (0.292)
**QUS**					
**SOS (m/s)**	1585 (25)	1578 (34)	1588 (34)	1583 (35)	0.252 (0.124)
**BUA (dB/MHz)**	121.1 (15.8)	114.3 (14.7)	116.3 (15.5)	112.9 (14.4)	**0.035** (0.118)
**SI**	104.8 (13.6)	97.5 (17.8)	102.4 (16.9)	(15.7)	**0.046** (0.065)

Values are reported as mean (SD). *p* value across all groups derived by ANOVA. *p* values adjusted for BMI, alcohol, smoking, physical activity. *p* < .05 was considered nominally significant and highlighted in bold. Those with pregnancies prior to baseline are not included in these analyses.

^a^Non-lactating (<2 mo): >15 mo (15-56 mo).

Abbreviations: BUA, broadband ultrasound attenuation; SI, stiffness index; SOS, speed of sound.

Regardless of cumulative duration of breastfeeding, there were no differences in endpoint BMD at the FN, TH, or spine compared with the non-lactating group. Apparent differences in TB were lost with adjustment including number of children (Table 4). BMD did not differ in those who were nulliparous (*p* values, .20–1.00) and, furthermore, there was no difference in BMD at any site between the nulliparous (*n* = 165) and non-lactating (*n* = 28) groups (*p* values, .29-.98).

### BMD change between age 25 and 35 in relation to pregnancy and breastfeeding

Being age 25 yr at baseline, the participants were assumed to have attained peak bone mass. Measured again at age 35 yr the trajectory of change in BMD over 10 yr in the complete cohort showed an overall loss at the FN and TH (FN ∆, -3.4%; TH ∆, -2.7%). In contrast, spine BMD appeared to be stable or even still accumulating, albeit by a small proportion (spine ∆, 0.9%) (Table 2A).

However, regardless of whether the women were parous or nulliparous, the percentage of BMD loss at the FN and TH did not differ, and furthermore, was similar regardless of the number of pregnancies. The net positive change in spine BMD was similar in parous, nulliparous, and multiparous groups (Table 5).

**Table 5:** Percentage BMD change (A) in women who were nulliparous or parous at age 35 and (B) by number of completed pregnancies during follow-up.

	**A. Nulliparous or had completed at least 1 pregnancy**			**B. Number of pregnancies completed during follow-up**			
**%ΔBMD at site**	**Nulliparous (*n* = 165)**	**Parous (*n* = 518)**	***p* value (*p*-adj)**	**1 Pregnancy (*n* = 144)**	**2 Pregnancies (*n* = 320)**	**≥3 Pregnancies (*n* = 54)**	***p* value (*p*-adj)**
**FN**	−3.3 (5.4)	−3.4 (6.3)	.849 (.480)	−3.4 (5.6)	−3.2 (6.8)	−4.4 (5.1)	.603 (.527)
**TH**	−2.7 (4.8)	−2.6 (5.7)	.895 (.123)	−2.5 (4.8)	−2.5 (6.3)	−3.9 (4.1)	.390 (.199)
**LS**	0.7 (5.2)	1.0 (4.6)	.489 (.191)	0.3 (5.1)	1.3 (4.5)	0.7 (3.8)	.193 (.112)
**Total body**	0.07 (3.3)	0.02 (3.3)	.876 (.325)	−0.3 (3.9)	0.2 (3.1)	0.01 (2.4)	.634 (.172)
**Head**	4.7 (3.9)	7.4 (4.4)	**<.001 (<.001)**	5.7 (4.7)	8.1 (4.1)	7.8 (3.8)	**<.001 (<.001)**

Values are reported as mean (SD). *p* values across all groups derived by independent *t* test (A) and ANOVA including nulliparous (B). *p* values adjusted for BMI, alcohol, smoking, and physical activity. *p* < .05 was considered nominally significant and highlighted in bold. Those with pregnancies prior to baseline are not included in these analyses.

With breastfeeding, the net change in BMD was negative at the hip and FN and the percentage BMD loss increased with cumulative duration of breastfeeding, particularly beyond 15 mo, compared with non-lactating women (FN ∆, -4.3%; TH ∆, -3.7%). However, if excluding women with 6 mo or less from weaning to DXA, the between-group differences were diminished (Table 6). Again, at the spine there was a net positive change in BMD; however, accretion was less with extended breastfeeding (Table 6).

**Table 6:** Percentage BMD change categorized by cumulative duration of breastfeeding during follow-up, in all women and excluding women with 6 mo or less from weaning to DXA.

	**Cumulative duration of breast feeding**				
**%ΔBMD at site**	**Non-lactating** ^**a**^ **(*n* = 28)**	**2–6 mo (*n* = 85)**	**7–15 mo (*n* = 229)**	**>15 mo** ^**a**^ **(*n* = 180)**	***p* value (*p*-adj)**
**All women**					
**FN**	−2.5 (5.2)	−1.6 (5.2)	−3.4 (5.3)	−4.3 (7.6)	**.011 (.005)**
**TH**	−1.5 (4.5)	−1.1 (4.7)	−2.5 (4.9)	−3.7 (7.1)	**.005 (.003)**
**LS**	0.7 (4.5)	1.9 (5.1)	0.9 (4.7)	0.6 (4.4)	.189 (**.008**)
**Total body**	1.9 (2.5)	1.0 (3.6)	−0.2 (3.4)	−0.5 (2.8)	**<.001** (**<.001**)
**Head**	9.5 (3.7)	7.1 (4.5)	7.2 (4.8)	7.5 (3.8)	.067 (<**.001**)
**Women with 6 mo or less from weaning to DXA excluded**	*n* = 28	*n* = 84	*n* = 187	*n* = 123	
**FN**	−2.5 (5.2)	−1.6 (5.2)	−2.6 (4.7)	−3.4 (8.6)	.229 (.205)
**TH**	−1.5 (4.5)	−1.1 (4.7)	−1.9 (4.6)	−3.1 (7.7)	.073 (.078)
**Spine**	0.7 (4.5)	1.8 (5.0)	1.5 (4.5)	1.1 (4.4)	.585 (.157)
**Total body**	1.9 (2.5)	1.0 (3.6)	0.4 (3.1)	−0.2 (2.7)	**.003 (<.001)**
**Head**	9.5 (3.7)	7.2 (4.5)	8.1 (4.5)	7.9 (3.7)	.085 **(.003)**

Values are reported as mean (SD). *p* values across all groups derived by ANOVA. *p* values adjusted for BMI, alcohol, smoking, physical activity, and number of pregnancies. *p* < .05 was considered nominally significant and highlighted in bold. Those with pregnancies prior to baseline are not included in these analyses. Women with 6 mo or less of weaning: *n* = 100 (1, 42, 57, in each breastfeeding group, respectively).

^a^Non-lactating (<2 mo): >15 mo (15-56 mo).

BMI differed between groups, BMI was lower with longer cumulative breastfeeding duration (*p* < .001), and the change in BMI over time (ie, between age 25 and 35) decreased with increasing duration (non-lactating BMI units, 2.6; lactating 2-6 mo, 2.5; 7-15 mo, 1.6; and ≥15 mo, 1.3; *p* = .002). The finding was unchanged when adjusting for number of children (*p* = .006).

### Recovery after weaning

**Figure 2:**
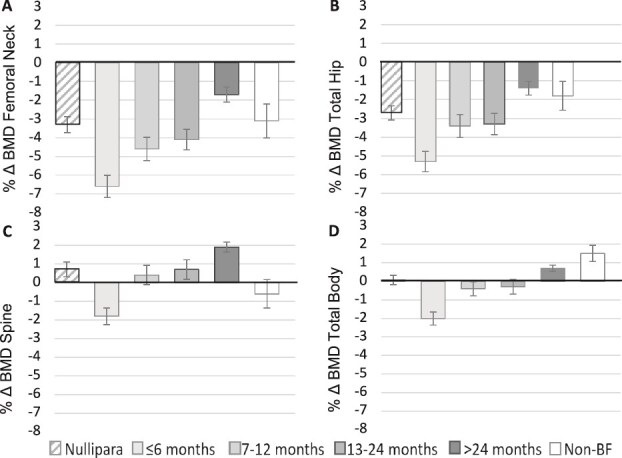
(A–D) BMD recovery, change in BMD based on time between weaning and DXA including non-breastfeeding (Non-BF) and nulliparous (Nullipara) women. Error bars represent SEM.

We also investigated the time frame of BMD recovery after cessation of breastfeeding. The median time from weaning of the last child until DXA was 25 mo (IQR, 9-48 mo). Women weaning within 6 mo before DXA had 6.6% lower FN BMD, but the loss of BMD was abrogated as a longer time from weaning elapsed; those who had weaned more than 24 mo previously had 1.7% lower BMD (*p* < .001). The result was regardless of the number of pregnancies (Table 7; Figure 2). At the spine, a clear stepwise accretion in BMD was apparent with a longer time elapsing between events.

**Table 7:** Percentage BMD change (A) based on time between weaning and DXA, (B) in non-lactating women, and (C) in nulliparous women.

	**A. Time between weaning and DXA**				B. Non-lactating	C. Nulliparous	***p* value (*p*-adjusted)**	
**%ΔBMD at site**	**≤ 6 mo (*n* = 93)**	**7–12 mo (*n* = 69)**	**13-24 mo (*n* = 80)**	**>24 mo (*n* = 283)**	**(*n* = 34)**	**(*n* = 165)**	**≤6 mo vs >24 mo**	**>24 mo vs nulliparous**
**FN**	−6.6 (5.6)	−4.6 (5.1)	−4.1 (4.9)	−1.7 (6.8)	−3.1 (5.2)	−3.3 (5.4)	**<.001 (<.001)**	**.033** (.396)
**TH**	−5.3 (5.0)	−3.4 (5.1)	−3.3 (5.0)	−1.4 (6.1)	−1.8 (4.4)	−2.70 (4.8)	**<.001 (<.001)**	**.026** (.247)
**LS**	−1.8 (4.3)	0.4 (4.3)	0.7 (4.7)	1.9 (4.6)	0.6 (4.4)	0.7 (5.2)	**<.001 (<.001)**	**.019** (.777)
**Total body**	−2.0 (3.4	−0.4 (3.1)	−0.3 (3.5)	0.7 (2.9)	1.5 (2.6)	0.07 (3.3)	**<.001 (<.001)**	**.017** (.985)
**Head**	5.3 (4.3	6.6 (3.7)	7.7 (4.5)	8.2 (4.3)	8.5 (4.4)	4.7 (3.9)	**<.001 (<.001)**	**<.001** (.721)

Values are reported as mean (SD). *p* values across 2 groups derived by independent *t* test. *p* values adjusted for BMI, alcohol, smoking, physical activity, and number of pregnancies. *p* < .05 was considered nominally significant and highlighted in bold. Those with pregnancies prior to baseline are not included in these analyses.

### Additional analysis

We further observed that head BMD was higher with increasing number of pregnancies (*p* = .020, *p*_adj_ = .011) (Table 4), with post hoc analysis indicating differences between more than 3 pregnancies and the nulliparous group. Similarly, the positive net change in BMD showed that parous women had a higher accretion of bone at the head (*p* < .001), even with increasing number of completed births (Table 5). With breastfeeding, the net positive change in BMD was observed after adjustment including number of pregnancies (*p* = .403, *p*_adj_ = .001), with values approximately 2% higher than in the nulliparous group (7.0 vs 4.7) (Table 6). After weaning, stepwise accretion in BMD was apparent with a longer time elapsing between cessation of breastfeeding and DXA (Table 7).

Pregnancy and breastfeeding were not associated with differences in bone microarchitecture, as measured by calcaneal QUS (Tables 2–4).

The data also indicate that, even for women who had pregnancies at an early age (ie, prior to baseline, *n* = 71), peak BMD (ie, measured at age 25) was attained, with parous and nulliparous women having similar values at the hip and spine. Furthermore, BMD at age 35 or net change in BMD was not different from those who were still nulliparous or had pregnancies after follow-up (*p*-value range, .530-.949) (Table S3).

## Discussion

This study of almost 700 25-yr-old women allowed BMD to be followed from the age of assumed peak bone mass, through to age 35 yr to investigate the effects of parity and lactation on the “normal” skeletal development at this age. The study showed that, overall, there are no detrimental effects on the skeleton. Pregnancy per se is not associated with differences in BMD and, while breastfeeding does result in bone loss at the hip, our 10-yr follow-up data indicate that this is transitory. Despite fluctuations in BMD from pregnancies and breastfeeding, recovery is complete, such that at age 35 bone density was equivalent to that of identically aged childless women. The implication of these findings can be used to reassure women that neither pregnancy nor breastfeeding over many months will adversely affect bone health.

Parity, regardless of number of pregnancies, had no negative impact on the skeleton; indeed, at the spine, BMD tended to be higher. This is in keeping with the literature, with most studies showing either a neutral or positive effect of pregnancy on BMD, and as such, similar to Paton et al,^14^ who found higher BMD in parous women compared with nulliparous women in a twin study. This was valid even for women who had had early pregnancies, before the baseline investigation and in line with data from the Third National Health and Nutrition Examination Survey (NHANES III), also prior to peak bone mass.^15^ A few prospective, albeit small, studies on pregnancy and BMD suggest a negative association,^4^^,^^16–18^ although these were all conducted in young women with a follow-up time close to the end of lactation and suggest a transitory loss of bone. In these studies, it is difficult to differentiate between effects resulting from pregnancy and those from lactation since the biggest losses were in women whose BMD assessment was early postpartum. In studies where a much longer time has elapsed from pregnancy to investigation (ie, women many years after menopause), parity or lactation has not been found to be detrimental to either BMD or risk of fracture.^8^^,^^9^ Taken together, the interpretation based on our findings and those of others suggests that other factors, beyond reproductive, are likely to be of greater importance for later fracture risk.

In our cohort, BMD at age 35 yr was not substantially different between women who breastfed, were non-lactating, or nulliparous. Even the cumulative duration of breastfeeding did not make a difference in the endpoint BMD. Nevertheless, breastfeeding does result in periods of net loss and an extended duration of breastfeeding, particularly beyond 15 mo, was associated with losses of approximately 4% at hip sites. The follow-up period of 10 yr for many study participants, including those with more than 1 pregnancy/lactation period, makes it difficult to determine the exact time of skeletal recovery. Nevertheless, the data demonstrate that whatever fluctuations in BMD have occurred across 1 or multiple pregnancies, the skeleton recovers. The skeleton’s ability to respond to the physiological demands placed on it is remarkable in terms of the quick bone homeostatic response to pregnancy and lactation and indeed to the cessation of lactation. Despite losses of as much as 7.6% at the spine and 3.6% at the FN in the very early days postpartum^16^^,^^17^—which are considerably higher than the losses observed post-menopause—recovery follows. The estimated rate of 0.5%-2% per month is greater than may be observed with pharmacotherapy for osteoporosis.^19^

DXA measurement in the PEAK-25 cohort was not timed to reflect reproductive events such as weaning and, compared with existing studies, this study had a longer postpartum to DXA measurement interval—up to 36 mo from the last pregnancy and median time from weaning of 25 mo. We see that recovery of the skeleton after weaning requires approximately 2 yr at the hip and FN, during which there is a plateau (or very slow accrual) during 7-24 mo. At the spine, consistent with other studies,^16^^,^^17^ recovery appears to be faster, as one would expect from more metabolically active trabecular bone, with clear stepwise accretion of BMD.

In this cohort, change in BMD across 10 yr of follow-up provides age-specific information on the trajectory of BMD in young women during these important years. In the cohort, we see that already by age 35, not long after peak bone mass is reached, bone density is already decreasing at cortical-rich sites, the TH and FN, while by contrast, bone is still accruing at the LS. It is well known that different skeletal sites reach peak bone mass at different ages, but there are almost no data on the skull; a cross-sectional study estimates continued increases up to the age of 50 yr at a rate of 0.1% per year through periosteal apposition.^20^ Our data suggest that the increases may be higher in parous women, but we can offer no obvious explanation for this.

In the present study, BMD was assessed using DXA, a technique that cannot differentiate between cortical and trabecular bone. Existing studies using HRpQCT^21^^,^^22^ indicate that pregnancy and lactation may be associated with differences in bone microarchitecture, a finding not reproduced in this study based on calcaneal QUS, and recognizing the limitations of this method.

To our knowledge, this is the largest prospectively followed cohort investigating the association between pregnancy and breastfeeding and related time frames on skeletal health. The results are likely to be robust based on the cohort size, the same age, and single center (reducing confounding from environmental changes, including differences in healthcare recommendations) and the comparison with non-lactating and nulliparous groups. Reassuringly, our data indicate that postpartum losses are transitory and that the overall change in BMD between ages 25 and 35 was no different from those who had never had children. Furthermore, this implies that adjustment for parity and lactation is not necessary in association studies.

An additional strength is that the participation rate was comparatively high and the only difference between those attending follow-up or not was a higher BMI in non-attenders; hence, selection bias is minor. We acknowledge that our data from Sweden, which has a high socioeconomic status and low average number of children, mean that our findings may not be extrapolated to cohorts/countries where nutrition or maternal healthcare may not be optimal or breastfeeding regimes are substantially different (65% at 6 mo/child).^23^ This could explain, at least in part, some of the inconsistencies in the literature.^24^^,^^25^

Undoubtedly, it is difficult to separate the effects from pregnancy and from lactation on BMD, without randomizing new mothers into lactating or non-lactating groups. Although information on breastfeeding was self-reported, the potential risk of recall bias was minimized because the cohort was young and the pregnancies relatively recent, such that recall is likely accurate since women have a strong emotional memory from childbearing.^26^ No information was available on breastfeeding intensity or whether breastfeeding was exclusive or mixed. While this could be a confounder, we consider it unlikely to make a major difference. Ultimately, our data, based on a substantial sample size, indicate that BMD will recover.

In conclusion, despite repeated fluctuations in BMD resulting from the physiological demands of multiple pregnancies and periods of breastfeeding, BMD recovers and ultimately does not differ from that of identically aged women without children. These findings are reassuring for young women.

## Supplementary Material

ms_pregnancy_lactation_BMD_Supplemental_material_zjaf087

## Data Availability

Data are available upon specific request to the authors.
