# Supplementary material for: Recovery of BMD after pregnancy and breastfeeding—a 10-yr prospective observational study of 25-yr-old women
Source: J Bone Miner Res. 2025 Jun 24;40(12):1360–9. doi: 10.1093/jbmr/zjaf087 (PMC12685719; doi:10.1093/jbmr/zjaf087)
Supplement: ms_pregnancy_lactation_BMD_Supplemental_material_zjaf087 [file ms_pregnancy_lactation_bmd_supplemental_material_zjaf087.docx]

# **Supplemental material**

**S-Table 1**. Baseline characteristics of women who participated or did not participate at 10-year follow-up

|  | **Participated**  **at 10-year follow-up** | **Did not participate**  **at 10-year follow-up** | **p-value** |
| --- | --- | --- | --- |
|  | *n=791* | *n=253* |  |
|  |  |  |  |
| BMI (kg/m^2^) | 22.8 (3.7) | 23.7 (4.2) | **<0.001** |
| Alcohol (g/week) | 44 (51) | 50 (148) | 0.381 |
| Current smoker | 25% | 31% | 0.094 |
|  |  |  |  |
| Parous PRIOR to baseline | 6% | 11% | **0.003** |
|  |  |  |  |
| Femoral neck BMD (g/cm^2^) | 1.056 (0.11) | 1.046 (0.13) | 0.282 |
| Total hip BMD (g/cm^2^) | 1.063 (0.12) | 1.057 (0.11) | 0.564 |
| Spine BMD (g/cm^2^) | 1.239 (0.13) | 1.240 (0.12) | 0.588 |
|  |  |  |  |

**S-Table 2.** BMD values based on Time from weaning until end-point DXA measurement (age 35)

|  | **BMD age 25** | **BMD age 35** | **ΔBMD (95%CI)** | **Δ%** | **P-value** |
| --- | --- | --- | --- | --- | --- |
|  |  |  |  |  |  |
| **≤ 6 months** | *n=93* | *n= 92* |  |  |  |
| Fem Neck | 1.061 (0.12) | 0.981 (0.10) | -0.080 (0.065-0.095) | -7.5 | **<0.001** |
| Total hip | 1.057 (0.11) | 0.993 (0.10) | -0.064 (0.050-0.77) | -6.1 | **<0.001** |
| Spine | 1.244 (0.12) | 1.228 (0.12) | -0.015 (0.003-0.028) | -1.3 | **0.015** |
| Total body | 1.174 (0.07) | 1.149 (0.09) | -0.025 (0.016-0.034) | -2.1 | **<0.001** |
| Head | 2.296 (0.25) | 2.434 (0.30) | 0.138 (-0.161-0.114) | 5.7 | **<0.001** |
|  |  |  |  |  |  |
| **7-12 months** | *n=68* | *n=67* |  |  |  |
| Fem Neck | 1.078 (0.15) | 1.020 (0.14) | -0.058 (0.041-0.074) | -5.4 | **<0.001** |
| Total hip | 1.083 (0.13) | 1.039 (0.13) | -0.044 (0.028-0.060) | -4.1 | **<0.001** |
| Spine | 1.270 (0.12) | 1.276 (0.13) | 0.006 (-0.021-0.010) | 0.5 | 0.471 |
| Total body | 1.186 (0.07) | 1.182 (0.8) | -0.005 (-0.005-0.014) | -0.3 | 0.354 |
| Head | 2.345 (0.21) | 2.519 (0.25) | 0.174 (-0.198-0.150) | 6.9 | **<0.001** |
|  |  |  |  |  |  |
| **13-24 months** | *n=78* | *n=78* |  |  |  |
| Fem Neck | 1.062 (0.11) | 1.013 (0.11) | -0.048 (0.035-0.061) | -4.6 | **<0.001** |
| Total hip | 1.065 (0.12) | 1.025 (0.12) | -0.040 (0.026-0.055) | -3.8 | **<0.001** |
| Spine | 1.262 (0.13) | 1.269 (0.13) | 0.007 (-0.021-0.007) | 0.6 | 0.332 |
| Total body | 1.169 (0.07) | 1.165 (0.09) | -0.004 (-0.006-0.014) | -0.3 | 0.438 |
| Head | 2.282 (0.20) | 2.471 (0.27) | 0.189 (-0.215-0.164) | 7.6 | **<0.001** |
|  |  |  |  |  |  |
| **≥25 months** | *n=249* | *n=247* |  |  |  |
| Fem Neck | 1.051 (0.12) | 1.030 (0.12) | -0.021 (0.012-0.030) | -2.0 | **<0.001** |
| Total hip | 1.063 (0.12) | 1.045 (0.12) | -0.018 (0.010-0.026) | -1.7 | **<0.001** |
| Spine | 1.235 (0.14) | 1.260 (0.14) | 0.025 (-0.032-0.017) | 2.0 | **<0.001** |
| Total body | 1.169 (0.07) | 1.180 (0.09) | 0.011 (-0.15-0.006) | 0.9 | **<0.001** |
| Head | 2.275 (0.23) | 2.478 (0.26) | -0.203 (-0.216-0.190) | 8.2 | **<0.001** |
|  |  |  |  |  |  |
| **Non-lactating** | *n=36* | *n=28* |  |  |  |
| Fem Neck | 1.060 (0.14) | 1.029 (0.11) | -0.032 (0.007-0.057) | -2.9 | **0.015** |
| Total hip | 1.078 (0.11) | 1.058 (0.12) | -0.019 (-0.004-0.043) | -1.9 | 0.101 |
| Spine | 1.234 (0.11) | 1.243 (0.11) | 0.010 (-0.032-0.013) | 0.7 | 0.407 |
| Total body | 1.190 (0.07) | 1.212 (0.08) | 0.023 (-0.034-0.011) | 1.8 | **<0.001** |
| Head | 2.351 (0.22) | 2.594 (0.23) | -0.242 (-0.277-0.208) | 9.4 | **<0.001** |
|  |  |  |  |  |  |

*Values reported as mean (SD). P-value across all groups by paired samples T-test. P<0.05 was considered nominally significant and highlighted in bold. BMD is unadjusted. Those with pregnancies prior to baseline are not included in these analyses.*

**S-Table 3.** Characteristics of PEAK-25 women who were parous prior to baseline (and therefore not included in the primary analyses)

|  | **Women parous prior to baseline** | |
| --- | --- | --- |
|  | **Baseline** | **Follow-up** |
|  | *n = 71* | *n = 41* |
| Age (years) | 25.5 ± 0.2 | 36.4 ± 0.8 |
| Height (cm) | 166.4 ± 6.0 | 167.3 ±5.6 |
| Weight (kg) | 67.4 ± 13.9 | 70.3 ± 12.6 |
| BMI (kg/m^2^) | 24.4 ± 5.1 | 25.1 ± 4.5 |
| Dietary calcium intake (mg) | 620 ± 393 | 534 ± 236 |
| Education level - Primary/secondary | 66 (94%) | 27 (60%) |
| Education level - University | 4 (6%) | 18 (40%) |
| Smoker - current | 32 (44%) | 9 (20%) |
| Smoker - non/previous | 41 (56%) | 36 (80%) |
| Alcohol g/week (median IQ) | 16 (6; 33) | 28 (9; 59) |
| Physical activity - high | 13 (18%) | 16 (22%) |
| Menstruation cycle – abnormal^a^ | 19 (26%) | 10 (22%) |
| Reproductive difficulties | - | 0 (0%) |
| Conditions associated with osteoporosis^b^ | 3 (4%) | 4 (11%) |
| Eating disorder | - | 6 (17%) |
|  |  |  |
| N^o^ of completed pregnancies |  |  |
| One | 58 | 11 |
| Two | 13 | 22 |
| Three or more (3-5) | 2 | 8 |
| Total N^o^ of children (mean N^o^) |  | 1.4 (0.97) |
| Time from last pregnancy to DXA (median) | - | - |
|  |  |  |
| N^o^ breastfeeding | 80% (57/71) | 90% (4/37) |
| Cumulative months of breast-feeding | 6.8 + 6.1 | - |
| Time from weaning to DXA (median) | - | - |
|  |  |  |
| DXA BMD (g/cm2) |  |  |
| Femoral neck | 1.042 (0.12) | 1.004 (0.13) / Δ -4,1% |
| Total hip | 1.051 (0.12) | 1.027 (0.13) / Δ -2.4% |
| Spine | 1.242 (0.14) | 1.250 (0.16) / Δ 0.3% |
| Total body | 1.176 (0.09) | 1.189 (0.11) / Δ 0.6% |
| Head | 2.360 (0.23) | 2.503 (0.25) / Δ 5.4% |
|  |  |  |
| T-score |  |  |
| Femoral neck | 0.513 (0.99) | -0.245 (0.92) |
| Total hip | 0.424 (1.03) | 0.154 (1.04) |
| Spine | 0.351 (1.15) | 0.417 (1.363) |
|  |  |  |

*Values reported as mean(SD); number(%); median(range); mean(range). ^a^Irregular or skipping >1 periods/yr. ^b^Inflammatory bowel disease, gluten intolerance, rheumatoid arthritis, cancer, osteoporosis or taking cortisone tablets ≥ 3 months.* ^c^*Daily calcium intake from milk, cheese, yogurt.*
